# Atomic Force Microscopy as a Tool Applied to Nano/Biosensors

**DOI:** 10.3390/s120608278

**Published:** 2012-06-14

**Authors:** Clarice Steffens, Fabio L. Leite, Carolina C. Bueno, Alexandra Manzoli, Paulo Sergio De Paula Herrmann

**Affiliations:** 1 National Nanotechnology Laboratory for Agribusiness (LNNA), Embrapa Instrumentation, P.O. Box 741, 13560-970, São Carlos, SP, Brazil; E-Mails: clarice@cnpdia.embrapa.br (C.S.); alexandra@cnpdia.embrapa.br (A.M.); 2 Departament of Biotechnology, Federal University of São Carlos (UFSCar), 13565-905, São Carlos, SP, Brazil; 3 Department of Physics, Mathematics and Chemistry (DFMQ), Federal University of São Carlos (UFSCar), 18052-780, Sorocaba, SP, Brazil; E-Mail: carolcastrob@gmail.com

**Keywords:** atomic force spectroscopy, atomic force microscopy, nanotechnology, nanoscience, nanosensors

## Abstract

This review article discusses and documents the basic concepts and principles of nano/biosensors. More specifically, we comment on the use of Chemical Force Microscopy (CFM) to study various aspects of architectural and chemical design details of specific molecules and polymers and its influence on the control of chemical interactions between the Atomic Force Microscopy (AFM) tip and the sample. This technique is based on the fabrication of nanomechanical cantilever sensors (NCS) and microcantilever-based biosensors (MC-B), which can provide, depending on the application, rapid, sensitive, simple and low-cost *in situ* detection. Besides, it can provide high repeatability and reproducibility. Here, we review the applications of CFM through some application examples which should function as methodological questions to understand and transform this tool into a reliable source of data. This section is followed by a description of the theoretical principle and usage of the functionalized NCS and MC-B technique in several fields, such as agriculture, biotechnology and immunoassay. Finally, we hope this review will help the reader to appreciate how important the tools CFM, NCS and MC-B are for characterization and understanding of systems on the atomic scale.

## 1. Introduction to Nanosensors

In order to promote a stable adsorption of molecules on microcantilevers, the Chemical Force Microscopy (CFM) technique is used here. CFM helps the molecules to “fall into place” in a spontaneous association in such way that they form a structurally well-defined aggregate. Thus, the purpose of the use of CFM in the construction of nanosensors is to achieve the organization of the molecules, to promote an orientation of functional groups, to contribute to chemical and physical stability of adsorption molecules to turn the device reproducible and sensitive.

Recent advances in the design and development of these sensors have led to simple microelectromechanical systems that can be manufactured easily, produced on a large scale and are capable of detecting very small mechanical deflections. The spring constant of a microcantilever ranges from 10^−3^ to 10^1^ N/m, enabling it to detect very small forces (10^−12^ to 10^−9^ N) [1]. These systems allow a fast response, low cost and the construction of arrays of sensors of small dimensions, thus enabling the investigation of microenvironments [2]. Microcantilevers are usually prepared on silicon and/or silicon nitride or polymeric materials, with dimensions from 100 to 500 microns in length from 0.5 to 5 micrometers in thickness, in the shape of a “V” (triangular) or “T” (rectangular) with a needle mounted on the free end [3].

Micro Electro Mechanical Systems (MEMS) are micro-electronic systems that incorporate chemical, magnetic and radiant heat micromechanical sensors and actuators. The first developments in the area of MEMS were performed in the 60s, systems were being commercialized by the 90s [4]. MEMS represent a family of diverse designs, devices with simple cantilever configurations, as used in atomic force microscopy (AFM), that are considered especially attractive as chemical and biological sensors. The ability of microcantilevers, to change their vibrational frequency or suffer deflection upon adsorbing molecules on their surface makes them excellent probes that can act as chemical, physical or biological sensors on nanoscale. Changes in vibrational frequency of micromechanical devices can be used to measure viscosity, density and flow rates in various systems. Deflections of the cantilever are due to the stress of molecular adsorption, which can be upward or downward depending on the type of chemical bonding of the molecule. In these systems, the change in frequency of the microcantilever has been reported to be proportional to the magnitude of the adsorbed mass [5–8].

## 2. Chemical Force Microscopy (CFM)

The chemical characterization of surfaces developed as an important technological tool allowing goods design and fabrication processes to fulfill high standards. This was achieved thanks to scientific advances in the atomic and molecular manipulation, the understanding of pathways for molecular binding but also to the conversion of such chemical information into reliable application methods.

In this context, Atomic Force Microscopy (AFM) can provide sensitive resources to measure and to map the surface chemistry information and to quantify the adhesive or repulsive forces associated to inorganic materials and biological samples, through the control of chemical interactions between the AFM tip and the sample [9–11]. Among AFM techniques, there is a very useful tool known as Chemical Force Microscopy (CFM), which is based on AFM tips chemically modified with specific exposed functional groups, carefully architected to carry out a specific function in a system [12–17]. Noy and coworkers have pioneered this technique, utilizing Lateral Force Microscopy (LFM) [18]. Applications to this tool include titration-AFM to obtain the apparent *Pka* value at the surface [10,15], determination of adhesive forces and energy on a surface, finding a specific substance by measuring single intermolecular forces (host-guest interaction in a complex environment), detection of chemical groups, determining surface heterogeneity, studying surface chemical reactions on the nanoscale and in real time [19]. All these applications found use in synthetic surface chemistry and in the creation of intelligent bioarrays, sensors, chips, and micro/nanofluidic devices.

Nanosensors are called smart devices due to their recognition selectivity and sensitivity. These crucial features are also typical characteristics of Chemical Force Microscopy, precisely used to address stabilization to a specific surface, since nanomaterials tends to aggregate, react chemically, or decompose if not treated properly. Here, it is worth mentioning that this treatment provided by CFM is known in the literature under several names, such as chemical derivatization, functionalization or modification. There are many ways to chemically modify surfaces and these modification methods involve addition, subtraction, or exchange of chemical groups or even restructuration of a molecule. For instance, we can cite the biochemical modification with enzymes [20], heat treatments, polarity change with corona discharge, plasmas, UV and gamma-radiations electron or ion bombardment, ozone [20–23], silylation [24,25] and lamination [26]. In the following, we review important parameters related to the CFM applications and its implications in nanobiossensors and nanomechanical cantilever sensors.

First of all, one of the major critical tasks in CFM is to deal with several parameters that can interfere in the measurements by causing noise in the signals collected and introducing artifacts into the results. Since CFM is based on an analytical probe consisting of a functionalized AFM tip, the quality of this probe is crucial. It has been observed that low quality tips can lead to artifacts in the images and consequently, to incorrect results [9]. It is also important to check the material the probe is made of, as well as if they have a suitable nominal tip radius, spring constant and resonant frequency that fits on our aim. More accurately the choice of the tip is made, more reliable and representative will be the results and, as consequence, they will be free of the inaccuracies that might be introduced by stiffness of the cantilever, the tip radius, shape, size and its dilation in AFM/CFM measurements.

Commercial tips may come with impurities, such as dust, organic residues or other common substances. Unclean probes can lead to contamination of the material under investigation, and because of that, before an experimental part begins, a cleaning step should be carried out in a UV/ozone chamber, to remove organic contaminants. This process will help to avoid artifacts in the final images and results. As previously mentioned, the tip shape can also significantly influence the results. If the tip has many things in contact with it (owing to large probe tip radius), during the surface scan, a lot of information will be lost. This effect can be explained by the direct relation between the radius of curvature of the tip and the tip-sample contact area [16]. The sharper is the tip; the higher will be the resolution and the confidence in the results. Scanning Electron Microscope (SEM) images of the tip are suggested here to determine the tip shape.

After optimizing CFM parameters, the surface functionalization is required. Firstly it is important to identify each substance, molecule or reagent that could be involved in the functionalization; secondly, this information should allow potential reactions and binding and eventually the complete molecular architecture to functionalize the tip to be predicted. This is crucial when CFM is used to measure adhesion and force (the tip surface chemistry must be well defined). For a clear understanding, each molecule, substance or atom (depending on the system) should be thought of as a small brick that will be used to build a wall. Here, some questions arise, such as: Should we lay this brick vertically or horizontally? Or is it better to put it at a specific angle? Is angle related to a binding site (active site)? How do we bind one brick to another? Is it necessary to use “cement” with specific characteristics? Should we control the solution pH? It is not important how many bricks are set on the tip surface, but it is important to know how to put them together. Chemical functionalization is a prerequisite for the firm attachment of the “bricks” in order to withstand the lateral force exerted during the scan acquisition and force measurements [12]. On balance, the functionalization of the AFM tip must be designed to obtain stability, sensitivity, and selectivity during the scan, in order to reduce the probability of non-specific bonding and the possible agglomeration of substances on the tip.

**Figure 1.**
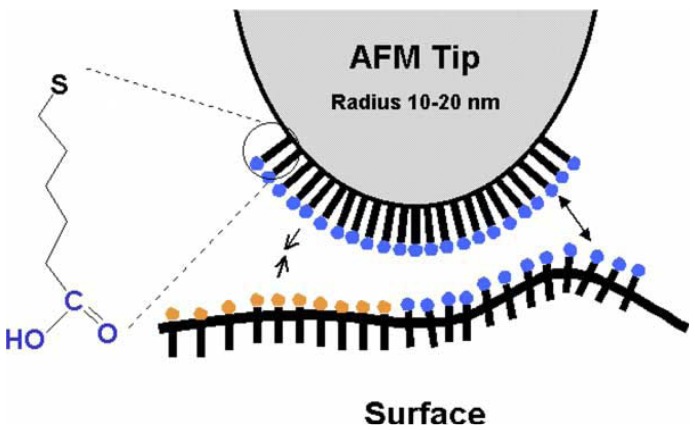
CFM principle: chemical tip modification with SAMs (Reprinted with permission [27]).

There are several ways to functionalize the AFM tip. Two of the commonest methods are known as mixing self-assembled monolayers (SAMs) and vacuum, thermal evaporation thermal vapor deposition and even, sputtering. The method of self-assembled monolayers (SAMs) is widely used to functionalize tips and surfaces. Terminal functional groups (-COOH, -CH3) are grafted onto alkane thiols that spontaneously form monolayers, under controlled conditions, on gold surfaces [9]. Organosiloxane monolayers (silanization) can also be formed on the tip [10]. The SAMs are usually applied by dropping reagents on the tip and then rinsing it after a specific time with another reagent (or ultra-pure water), or by the immersion of the tip into a specific solution (say in a beaker, like the Layer-by-Layer (LBL) process for thin films), followed again by washing. A typical result of SAM is depicted in Figure 1. Sometimes, the design of SAMs on a tip can follow the patterns of Nature; *i.e.*, mimicry can be an excellent source of inspiration. Self-assembled monolayers offer new opportunities to increase the fundamental understanding of self-organization structure-property relationship, and interfacial phenomena [3]. SAMs can provide the needed design flexibility, as functionalized chains of polyethylene glycol (PEGs).

In vacuum/thermal vapor deposition thermal evaporation should be handled with care in order to prevent the breaking of the tip. To use this procedure, the physical and chemical conditions must be chosen carefully, as surrounding humidity and temperature, environment medium of analysis (air, liquid or vacuum) and time of incubation of reagents. Generally, this process must be done in a chamber (with vacuum or not), which chamber must be cleaned with an inert gas before the beginning of the functionalization. It is important to highlight that after the functionalization process, the probes should be gently dried in a stream of inert gas (argon is used most frequently), and stored under an atmosphere of the same gas, in a desiccator, to avoid high humidity and the oxidation of the probe. The sputtering techinique can be used to functionalize the AFM tip with a metal coating in order to induce specific properties to the tip. Some of these properties are: electric and thermal conduction, optical reflectivity and ferroelectricity [28].

Hereafter, some forms of analysis offered by CFM will be next discussed: measurement of contact angle (θ), force *vs.* distance curves, histograms, and adhesion maps.

**Figure 2.**
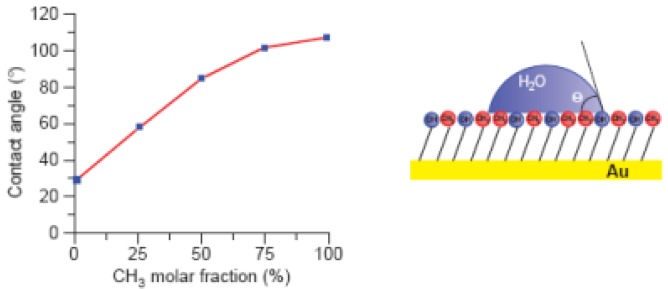
Chemical force microscopy (CFM): principle and application to the probing of hydrophobic forces. Water contact angle (θ) values measured on mixed self-assembled monolayers (SAMs) of CH_3_- and OH-terminated alkanethiols, plotted as a function of the molar fraction of CH_3_-terminated alkanethiols (reprinted from [29] with permission).

Contact angle is frequently used to characterize surface energy properties across the three-phase boundaries, where liquid, gas and solid phases meet [16], and to gain a better understanding of force/adhesion events related to apolar and polar components. θ represents the ability of a liquid to spread on a plane surface and it is measured as the angle between the outline tangent of a drop deposited on a solid and the surface of this solid [29–31]. In addition, contact angle can also be related to thermodynamic concepts and are measured by fitting a mathematical expression to the shape of the drop and then calculating the slope of the tangent to the drop at the three-phase boundaries interface line. Here is important highlight the Cassie's law. This law is derived from the thermodynamic definition of contact angle and concerns to the contact angle of a macroscopic droplet on a chemically heterogeneous surface and is successfully used to explain the superhydrophobicity and self-cleaning mechanism of various natural and artificial surfaces [32–34]. Examples of such analysis can be observed in Figure 2.

**Figure 3.**
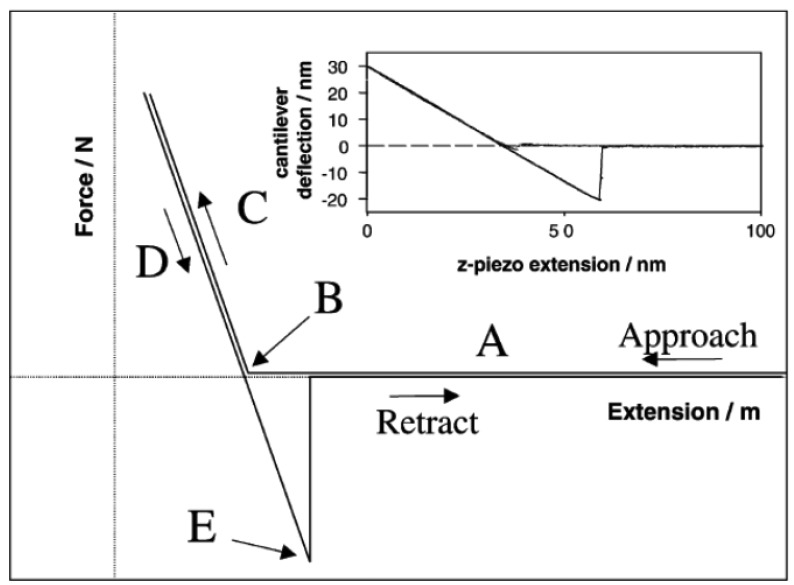
Force-displacement curves were formed. (**A**) tip is a far distance from the surface and there is no interaction; (**B**) the tip contacts the surface; (**C**) the cantilever is bent and a repulsive force (positive) is measured; (**D**) the cantilever holder is retracted from the surface and an adhesive (negative) interaction between the tip and surface is measured; (**E**) the pull-off point (reprinted from [9] with permission).

Force *vs.* distance curves (or force *vs.* displacement vertical curves) are used to collect quantitative and direct information on the force and adhesion forces between the AFM tip and the sample, including elongation, separation, elastic deformation (stretch), and binding forces. These curves can provide significant details of recognition events, homogeneity, chemical composition of the sample and biological events. Figure 3 briefly explains each part of a force *vs.* distance curve during the approach and retraction of the tip. Note that the force at point E is the pull-off force, defined as the highest force required separating the probe from the surface [27].

In order to confirm the results and quantify the differences between adhesion/repulsive force measurements and eliminate possible noise caused by artifacts, it is required to record a large number of force *vs.* distance curves: at least forty for every type of measurement. These data should be plotted as a histogram of the frequency (%) of the values observed for each interaction *vs.* the adhesion force (pN) [35]. These histograms show the statistical distribution of the adhesion forces, which are given by the vertical distance found in the force curve (point E to horizontal line).

**Figure 4.**
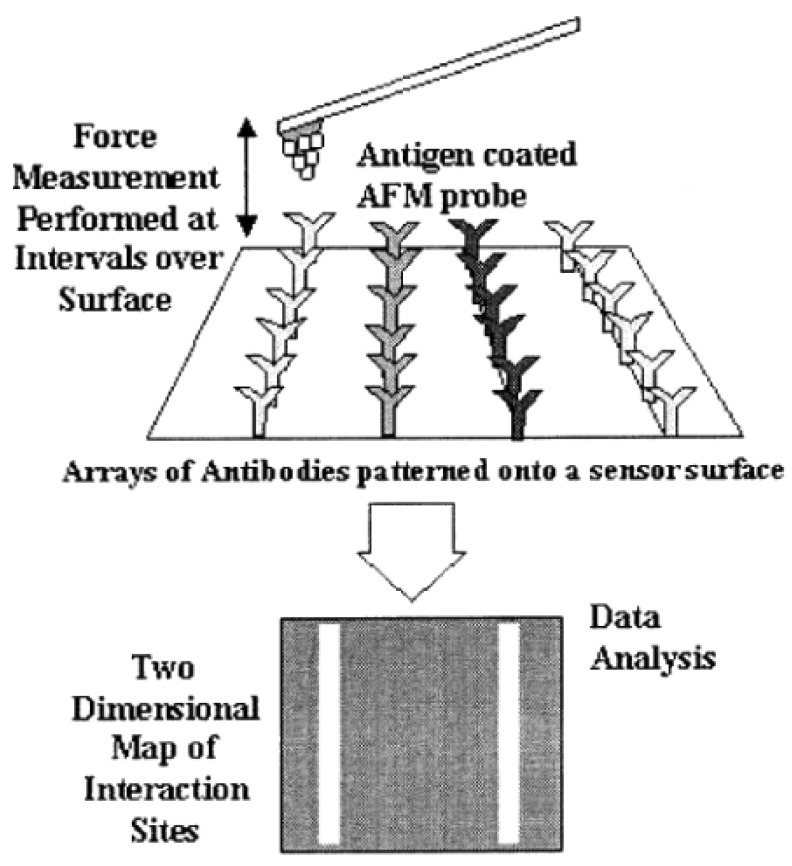
A schematic diagram of an antigen-coated probe mapping specific interaction sites on a substrate patterned with three antibody species (reprinted from [26] with permission).

Given that adhesive forces are directly linked to interactions between chemical functional groups on the tip and the sample surface [32], adhesion maps can give additional, and not less important, information with respect to the force *vs.* distance curves. As it can be seen in Figure 4, an adhesion map is a way of mapping surface forces with a very high spatial resolution (usually 10 nm) [9]. Here, the approach relies on combining information from the force *vs.* distance curves at each point (pixel) in an image field with simultaneously acquired topographic data to yield a high-resolution map of chemical interaction sites [9].

The white lines displayed on the adhesion force map (Figure 4) are a result of the specific interaction between the antigen in the tip and its specific antibody (light grey). Thus, as may be clear, an adhesion map is built as result of repeated specific interactions between the tip and the surface. Depending on the system studied, this repetitive process can damage the functional molecules on the tip apex during mapping, which can be a hard challenge to deal with [36]. Adhesion maps have another remarkable application: establishing a fingerprint for each molecule, substance, and receptor or complexation event in order to recognize and locate specific substances on a surface [37]. This could be a useful tool that enables surface differentiation in cutting-edge technologies [38]. A typical adhesion map is formed by pixels of specific recognition (differences detected in the deflection signal) [39,40].

The interpretation of adhesion maps and force curves can occasionally be difficult because of the surface roughness and elastic behavior of the sample. Sometimes it is necessary to process the primary results with statistical programs, depending, of course, on the form in which the AFM software outputs the results.

Additional physical techniques are used to support CFM data. Usually, these are traditional methods used to analyze the molecular and elemental composition of surfaces, such as X-ray Photoelectron Spectroscopy (XPS), Fourier Transformed Infrared Spectroscopy (FTIR) and Raman Spectroscopy. For instance, Raman spectroscopy provides additional information on the chemical composition of materials and, because of that, may confirm whether the tip was indeed functionalized [41,42]. The same is valid to FTIR and XPS, which provide, respectively, the molecular composition of the surfaces of materials and the measurement of the elemental composition of the surface [43,44]. Nevertheless, it is also worth mentioning that these cited techniques provide a spatial resolution limited by diffraction and should be used just as complementary information to the CFM studies. Additionally, all of these techniques can also be compared with Dynamic Molecular Model Data too, and used to verify what is predicted by these models.

Finally in CFM studies, it is important to make the building process of the nano(bio)sensor reproducible in order to make the CFM measurements reproducible. Because of that, the majority of the parameters involved in their construction and operation should be kept as constant as possible, such as surrounding humidity and temperature, environment medium of analysis (air, liquid or vacuum) and time of incubation of reagents, as previously indicated. Even small changes in the parameters that determine the chemical force interaction between the tip and the sample can lead to enormous changes in images, curves and results [21].

## 3. Nanomechanical Cantilever Sensors (NCS)

The detection of chemical species through sensors is one of the most intensely investigated fields of science and technology, owing to the importance and variety of applications. A chemical sensor is an instrument that, when exposed to a particular type of substance (the analyte), transforms the chemical information, such as polarity or difference in concentration, for example, into an analytically measurable signal, such as electrical resistance, conductivity, potential difference or frequency. This transformation is called signal transduction and is of central importance to the working of any sensor [33].

**Figure 5.**
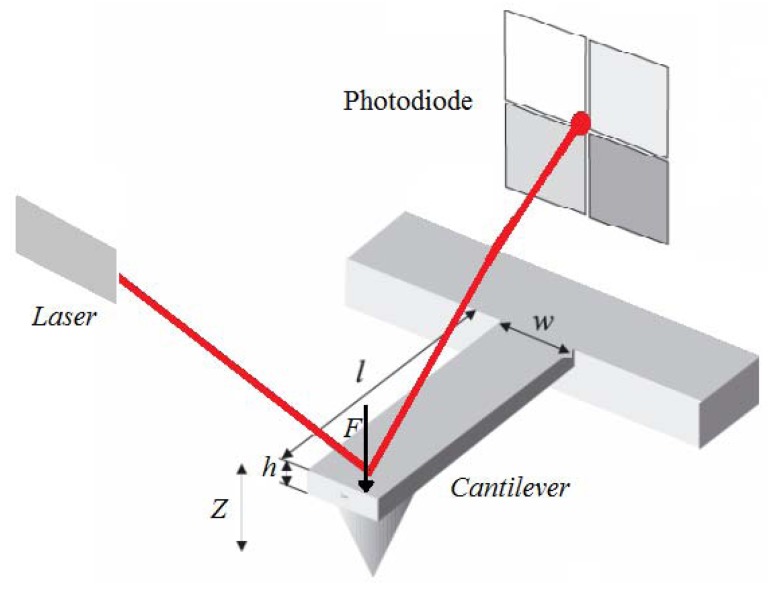
Illustration of the displacement of the cantilever (h, w and l, thickness, width and the length of the cantilever, respectively).

Microcantilevers were first used in Scanning Force Microscopy. The deflection of a cantilever can be measured with an optical sensor when it is a small deflection; the vertical displacement of the tip (Z) is directly proportional to the force on it (F) and is thus a direct measurement of the strength of interaction between the tip and a surface (Figure 5). The principle of operation of a cantilever sensor is based on the adsorption of analytes at the surface of the cantilever (coated with a sensing layer), which usually leads to an induced surface stress and an increase of the apparent mass of the cantilever [8–34]. The change in mass leads to deflection of the cantilever in the Z direction.

Treating the rectangular cantilever as a vibrating spring-mass system, its resonance frequency (f_res_) can be calculated as:

(1) $$f_{\text{res}}=\frac{1}{2\pi }\sqrt{\frac{k}{m}}$$

where k is the spring constant and m the mass.

The mass of a cantilever can be expressed as: m = ρ.h.l.w, where ρ is the density of the material, h its thickness, w its width and l the length.

The spring constant k is a function of the geometric and material parameters:

(2) $$k=\frac{E*w*h^{3}}{4*l^{3}}$$

where E is Young's modulus of the material in the cantilever. For example, for silicon (100) E = 1,3 × 10^11^ N/m^2^. The surface stress is uniform and acts on the surface, increasing (in the case of compressive stress) or decreasing (in case of tensile stress) the surface area. If this stress is not compensated at the opposite side of a cantilever, the whole structure will bend. A change in surface stress on only one side of the cantilever will cause a static deflection and the deflection can be calculated as:

(3) $$\Delta Z=\frac{3l^{2}(1-\upsilon )}{\text{E}*{\text{h}}^{2}}\Delta \sigma$$

where *υ* is the Poison rate (*υ* = 0.24 for a rectangular cantilever), δσ= σ_1_ − σ_2_ the difference in surface, σ_1_ and σ_2_ being the stress on the upper and lower side of the cantilever, respectively. Poisson's ratio is 0.24 for a rectangular cantilever and the difference in surface stress approximately follows Stoney's equation [45], which relates cantilever deflection (Z) to applied stress (σ).

The cantilevers can be operated in two modes static and dynamic. The static mode allows the analysis of cantilever bending in different ambient conditions to measure surface stress changes while, in the dynamic mode, the change in resonance frequency of the cantilever is monitored. Changes in surface stress result from the adsorption or electrostatic interactions between molecules on the surface, as well as changes in surface hydrophobicity and conformational changes in the adsorbed molecules [35].

When cantilevers are functionalized with sensitive materials such as metals, polymers, enzymes, thin layers, among others, the sensitive coating may interact with analyte molecules that selectively adsorb or bind by chemical affinity, converting the cantilever into a selective and sensitive sensor [36], which responds to specific substances or groups of substances.

The interaction between the analyte and the surface layer on the sensor can be reversible or irreversible. In the reversible case, the analyte interacts with the surface layer of the sensor to produce a response and when the analyte molecules are removed, the senor returns to its original state. In the irreversible case, the analyte undergoes a chemical reaction at the sensor surface catalyzed by the sensor material. Here, the analyte is consumed in the sensing process, although the number of molecules reacting is often a small proportion of the total number within the sample.

The method used in the functionalization has a strong influence on the sensitivity, because this depends on a number of parameters such as uniformity of the coating and the possibility of molecular reorganization by changing interactions due to external stimuli or analytes. The cantilever selectivity depends on the detection layer, which may be built according to principles of molecular recognition.

Several methodologies have been described in the literature for microcantilever surface modification with organic [37] or inorganic layers [39,40,46], some of which are described below. Functionalized cantilever bending may be due to the adsorption of gas, if the expansion coefficients of the materials on the two sides are different. The absorption of water vapor into an inorganic sensing layer has been investigated by Thundat *et al.* [39]. They coated one side of the silicon cantilever with a thin film of gelatin or phosphoric acid, as hygroscopic materials. The functionalization was carried out by sliding the cantilever into the solution until completely covered. The resonance frequency was measured in different conditions of relative humidity. The cantilevers functionalized with phosphoric acid (H_3_PO_4_) showed a decrease in the resonant frequency with the reduction of relative humidity due to the increasing effective mass, while the cantilevers coated with gelatin film showed an increase in the resonant frequency. Thundat *et al.* [40] also reported the deflection of commercially cantilevers, using a position sensitive detector. Silicon nitride cantilevers were supplied with 4 nm chromium and 40 nm gold layers and other cantilevers with 5–13 nm of aluminum on one surface. For the gold coated cantilever the deflection varied almost linearly and reversibly with changes in relative humidity, while in the uncoated cantilever, the deflection was negligible. The cantilevers with 5–13 nm of aluminum were very sensitive to changes in relative humidity. Pinnaduwage *et al.* [46] reported the detection of 10–30 parts per trillion of pentaerythritoltetranitrate and hexahydro-1,3,5-triazine within 20 s of contact with a silicon microcantilever whose gold surface had been modified by immersing the cantilever into a 6 × 10^−3^ M solution of 4-mercaptobenzoic acid, to build a self-assembled monolayer. The monolayer coating was shown to be quite stable for several months under normal operating conditions.

**Figure 6.**
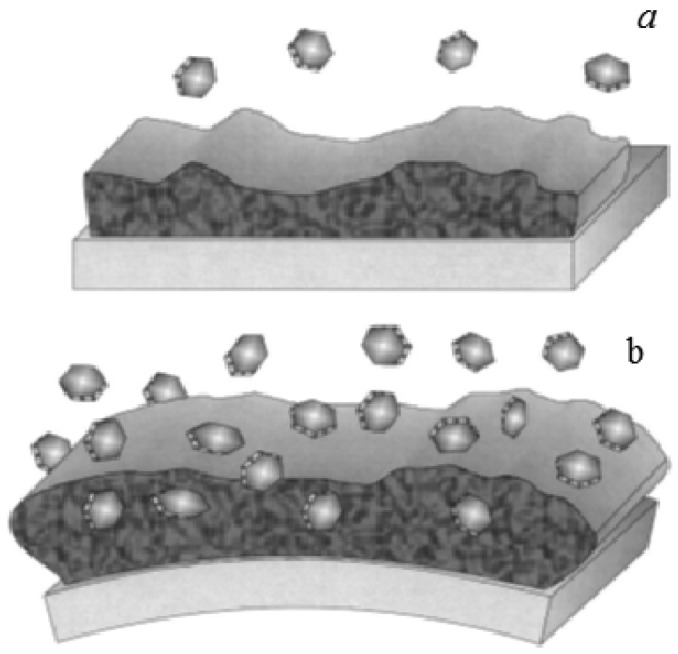
Sketch of the absorption-induced surface stress at the surface of cantilevers: (**a**) absorption and (**b**) penetration of target molecules into the sensing layer (reprinted from [38] with permission).

**Figure 7.**
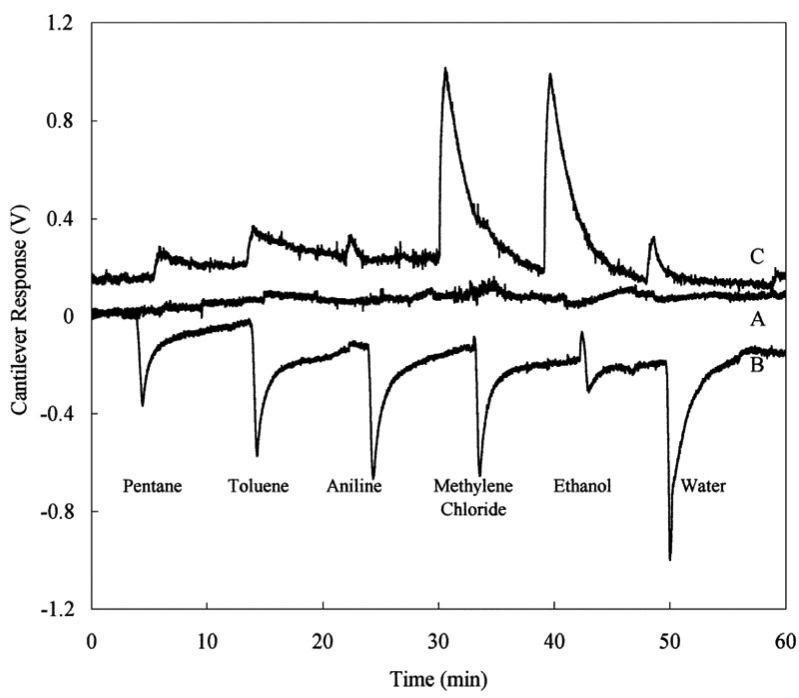
Cantilevers (**A**) treated with Aqua regia; (**B**) coated with gold, removed from one side by a focused ion beam and (**C**) coated with gold and thin films of polymeric chromatographic stationary phases, removed from one side a focused ion beam (reprinted with permission [48]).

Functionalizing the cantilever with polymer coating renders possible highly sensitive identification of gases and volatile organic compounds. The possibility of using different polymers allows one to functionalize the cantilevers with a selective coating to analyze mixtures of volatile organic compounds [47]. When cantilevers are functionalized with polymeric layers, they can absorb molecules of an analyte, causing a swelling of the polymer matrix and thus resulting in a differential cantilever stress. This expansion induced cantilever surface stress can occur in two different ways: target molecules can be adsorbed on to the functionalized surface (Figure 6(a)) or penetrate the sensing layer deposited on the surface of the cantilever (Figure 6(b)) [38]. Spin-coating of the polymers usually results in uniform films with controlled thickness, which is easily varied by changing the spin speed or switching to a different viscosity. However, in the literature there are divergences over the technique of spin-coating deposition. Spin-coating with polymer layers usually causes an unwanted deposition on the passive side of the microcantilever. On the other hand, microcantilevers were coated on one side by using a spin-coating technique by Betts *et al.* [48]. The difference in the resonance frequency between the coated and uncoated microcantilever is related to the change in the mass of the resonating structure. The results showed that the selectivity, as indicated by differences in relative responses to the test analytes, was different for the solvents phases which differed significantly in polarity (Figure 7).

Boisen *et al.* [49] have developed integrated piezoresistive read-out cantilevers and investigated their use as humidity and alcohol sensors. The cantilevers are silicon/silicon oxide layers with integrated polysilicon resistors. For application of cantilever based-sensors as humidity and alcohol sensors in water it was necessary coating one of the sides of the cantilevers with a water absorbing polymer. The humidity was controlled by mixing dry and wet nitrogen gas (2 to 60% of relative humidity) into a chamber. Although the response of the cantilever sensor to humidity change was not linear, it was reproducible. On the other hand, the alcohol sensor in water responded successfully to different amounts of alcohol, being the cantilever deflection proportional to alcohol entering the polymer and expelling water from the film. So, this sensor can be used to detect alcohol in water. Goericke and King [50] have reported finite element simulations of piezoresistive cantilevers. These authors investigated the sensitivity of piezoresistive cantilevers with respect to changes in cantilever length, width and thickness, and piezoresistor size, location, and depth. In the piezoresistive cantilever sensor, the cantilever width is an important parameter, which should be maximized for optimal sensitivity. The cantilever length, however, is not critical for high sensitivity. The sensitivity of piezoresistive cantilevers increases with decreasing piezoresistor thickness. The piezoresistor width need only be kept below 60% of the cantilever width for good sensitivity and the piezoresistor length should be minimized to reduce overall resistance and increase device sensitivity.

Then *et al.* [47] used a system of six differently polymer-coated cantilevers to analyze quantitatively and qualitatively a series of volatile organic compounds and a mixture of three different components (1-butanol, toluene and *n*-octane). The polymers were chosen to cover a wide range of polarities and different chemical behavior. The coating of the six different polymers (polydimethylsiloxane, polyetherurethane, poly(cyanopropylmethyldimethylsiloxane), phenylvinyl-polydimethylsiloxane, poly(phenylmethyldimethylsiloxane) and polyethyleneglycol) on the cantilevers was performed by micromanipulator, patch-clamp pipettes and spray-coating. The array of the six differently coated cantilevers together with principal component regression was capable of quantitative analysis of complex gas mixtures as showed for ternary mixture of 1-butanol, toluene, and *n*-octane. The interactions of a polar polymer with a polar analyte are much stronger and therefore the partition coefficient increases. Partition coefficient is the ratio of concentrations of a compound in the two phases of a mixture of two immiscible solvents at equilibrium. The polar 1-butanol with the ability of additional H-bonding shows up with the lowest detection limit (LOD), toluene which can be polarized as an aromatic system ranges in the middle and the non-polar n-octane has the highest LOD.

Dong *et al.* [51] assessed three polymer layers (polyethyleneoxide, polyvinylalcohol and polyethylenevinylacetate) on resonant microcantilever for the detection of volatile organic compounds. They coated the surface of the microcantilever with an air brush and pipettor. The polarity of the volatile compound strongly influenced its diffusivity in the polymer layer. It was observed that the most hydrophilic (polyvinylalcohol) and hydrophobic (polyethylenevinylacetate) polymers showed the lowest sensitivity to the least polar solvents (hexane and octane) and most polar solvents (ethanol and acetone), respectively.

**Figure 8.**
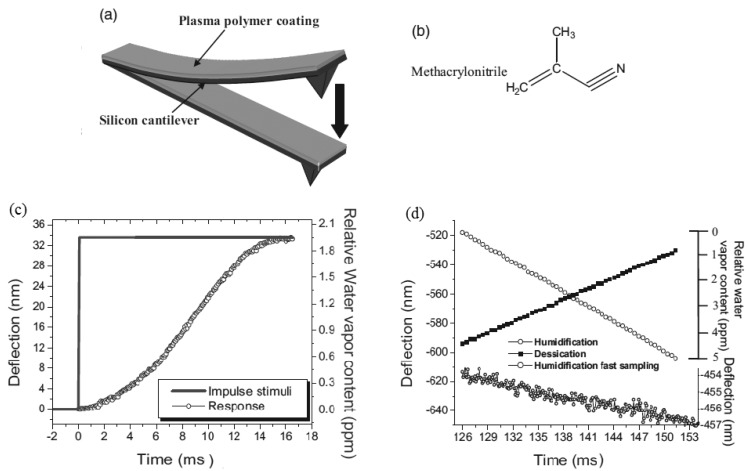
(**a**) Simplified diagram of the functionalization of the silicon cantilever coated with polymer; (**b**) Chemical structure of methacrylonitrile; (**c**) Deflection of cantilever in response to sudden humidity change and (**d**) Dynamic sampling during linear humidification and desiccation (reprinted with permission [52]).

Silicon microcantilevers for humidity detection were studied by Singamaneni *et al.* [52]. The cantilever with a low flexural rigidity was coated with a plasma-polymerized methacrylonitrile monolayer [Figure 8(a,b)] and it showed for instantaneous changes of humidity a response time of 9.5 ms in the range from 10 to 90% of humidity [Figure 8(c)]. It was observed a linear response in the ramps of humidification and desiccation (1% of relative humidity per second) showed in [Figure 8(d)], with resolution of ±10 ppb of water vapor. The authors suggested that the desirable responsive behavior of the bimaterial cantilever is associated with the integration of a crosslinked polymer, high internal stresses, and firm adhesion to the silicon.

Sub-ppm sensitivity and the response of piezoresistive cantilevers arrays coated with different polymers [polyvinyl alcohol (PVA), polyethylene imine, polyacryl amide and polyvinyl pyrrolidone] to detect various alkanes were investigates [53]. It was demonstrated that this array of sensors coated with different polymers had the selectivity of discriminated individual alkanes in a homologous series using principal component analysis of the pattern formed from the responses of polymer-coated cantilevers. Accordingly, the sensitivity of the piezoresistive cantilever was determined to be 0.05–0.13 ppm. The adsorption of analytes on the polymer layers induces surface stress due to swelling of the polymers, resulting in the bending of the cantilevers. The adsorption of water molecules on the surface of the cantilever increases its mass and thereby decreases its resonance frequency.

Electronic noses with arrays of cantilever sensors that respond individually to vapors can produce a distinguishable response pattern for each separate type of analyte or mixture. Their use opens up applications in many fields such as quality and process control, medical analysis, fragrance, oenology, and as sensing devices for volatiles compounds [54].

Baller *et al.* [54] used an artificial nose based on a microfabricated array of eight silicon cantilevers to detect analyte vapors. Each cantilever of the array was functionalized with a different polymer (polyvinylpyridine, polyurethane, polystyrene and polymethylmethacrylate). To observe the transduction of physical and chemical processes into deflection, the swelling of a polymer layer on the cantilever was monitored during exposure to the analyte. It was observed that the swelling process is related to the vapor pressure and the solubility characteristics of the analyte in the polymer. A standard recognition patterns was created upon exposure to known gaseous analytes, so that alcohols, acetone, dichloromethane, toluene and heptane could be identified.

Lang *et al.* [55] also evaluated the use of an artificial nose with a microcantilever sensor array to characterize solvent vapors and fragrances. All eight cantilevers were functionalized with a polymer layer by the inkjet spotting technique. The deflection was measured with a surface emitting laser and a position-sensitive detector received the optical beam. The cantilever deflection in response to solvent vapors and fragrances was caused by the diffusion of the molecules, resulting in a swelling of the polymer layers. When the cantilever was exposed to dry nitrogen, the beam returned to its initial position, demonstrating that the sensing process is reversible and reproducible.

## 4. Microcantilever-Based Biosensors (MC-B)

The great interest in developing microcantilever-based biosensors stems from the binding specificity of biomolecules for analytical sensing, combined with the unlimited ability of this device to transduce signals between the biomolecule and an electronic device. According to the International Union of Pure and Applied Chemistry (IUPAC) definition [56], a biosensor is a sensor possessing biological components (antibodies, enzymes, DNA), which detects their interaction with the analyte of interest by a dedicated transduction mechanism. The transducer is the device capable of converting the physical (e.g., resistance, voltage, conductivity) or chemical signals (electron transfer from a chemical reaction) into measurable, usually electronic, signals whose magnitude is proportional to the concentration of the species or chemical grouping.

The advantages of microcantilever-based biosensors are small size, label-free detection, and a potential for arrayed operation [57]. Instrumentation based on microcantilever arrays would be very attractive because it could detect multiple biomarkers simultaneously with high sensitivity and selectivity in small sample volumes [58].

Microcantilevers are micromechanical devices with dimensions on the order of 100 μm × 30 μm × 0.6 μm [6]. They can readily be fabricated on silicon wafers and other materials, but silicon microcantilevers are the most commonly used in biosensors. They are physical sensors that respond to surface stress changes due to chemical or biological processes [59]. These sensors can measure forces and stresses with extremely high sensitivity when fabricated with very small force constants [58]. Adsorption of molecules on one of the surfaces of the biomaterial cantilever results in a differential surface stress due to adsorption-induced forces, which is manifested as a deflection [58,60]. In addition to cantilever bending, the resonant frequency of the cantilever can be changed by mass loading [58,61]. These two types signal, namely the adsorption-induced cantilever bending when adsorption is confined to one side of the cantilever and the adsorption-induced vibrating frequency change due to mass loading, can be monitored simultaneously [40]. Resonant cantilevers immersed in liquid suffer from high damping losses and reduced sensitivity [62]. Thus, the resonant frequency operating mode of the cantilever is normally limited to the detection of gas-phase samples, while the bending mode can be used for both gas and liquid samples [57,62].

The commonest method used to measure cantilever displacement is the optical lever (see Figure 5) [63,64]. A focused laser beam is reflected off the cantilever surface, and captured by a PSD (position sensitive detector). The cantilever displacement causes movement of the laser spot on the PSD and hence a change in its output voltage [65]. In microcantilever biosensors, the accuracy of measurement depends strongly on accurate determination of the surface deflections. This optical lever approach is not suitable for a cantilever array, because the response of only one device can be captured. Custom-made arrays of lasers and PSDs for use with several cantilevers in parallel lead to greatly increased instrumentation complexity and difficulty of alignment [66]. The cantilever bending may also be sensed with a deflection sensor integrated into the cantilever [38]. A major advantage of this set-up is that it allows massively parallel arrays and can be performed without the need for optics. For deflection sensing with elements integrated into the cantilever, piezoresistive sensing is the most widespread approach [67], although there are others, including piezoelectric [68] or capacitive sensors [69]. The high piezoresistive coefficient of doped single-crystal silicon makes silicon piezoresistive sensors an attractive option [70].

The sensitivity of microcantilever biosensors can be increased by optimizing the geometry of the cantilevers and immobilization techniques. For adsorption-induced cantilever deflection, longer and thinner cantilevers with small force constants show higher sensitivity. However, larger area cantilevers show faster detection of low concentrations of target molecules. Thus, the optimal cantilever dimension will depend on the dimension of the cantilever chamber and the analyte delivery system [58]. The use of a reference cantilever basically improves sensitivity. New piezoresistive cantilevers designs have been developed that show less drift and higher signal-to-noise ratios. Selectivity of detection with microcantilever sensors in complex samples still remains to be solved [71].

The functionalization of the microcantilever surface is the most important step in the development of microcantilever-based biosensors. This step is critical to cantilever sensing because it determines the surface density of receptor molecules and thus the number of binding events on the active surface. It may also affect how efficient the transduction is from the biomolecular interaction to the change in surface stress on the cantilever. Additionally, an effective passivation can significantly reduce the nonspecific binding, so that the background signal can be minimized. Depending upon the final application of the device, various types of immobilization can be used. Generally, cantilevers are coated on one side with 2–3 nm of chromium and 25–30 nm of gold. Chromium acts as an adhesive layer for the gold. When the functionalization of the microcantilever surface involves silanization, both side of the cantilever is used; and when the coating involves thiol, only the gold side of the cantilever is used. For thiol self-assembled monolayers (SAMs) and organosilane modification, dip coating is the preferred method for functionalization, to allow high density immobilization on the cantilever surface. Thiol SAMs are self-limited to coverages of a monolayer of the thiol on a gold film. Silane coating can yield multilayered films upon extended exposure to the solution. Regardless of the type of coverage, it must be prepared no more than 48 h before analysis [58].

Since 1990, the technique of sensing by means of the bending response of microcantilevers is being increasingly used for the detection of biomacromolecules, rather than small molecules. A. Subramanian *et al.* [6] described a glucose oxidase-coated microcantilever for the specific detection and quantitation of glucose and they assessed the signal transduction mechanism. The enzyme was immobilized on one side of a silicon cantilever, coated with gold. The magnitude of the bending response of the glucose oxidase-derivatized microcantilever was concentration dependent over a large range of glucose concentrations.

**Figure 9.**
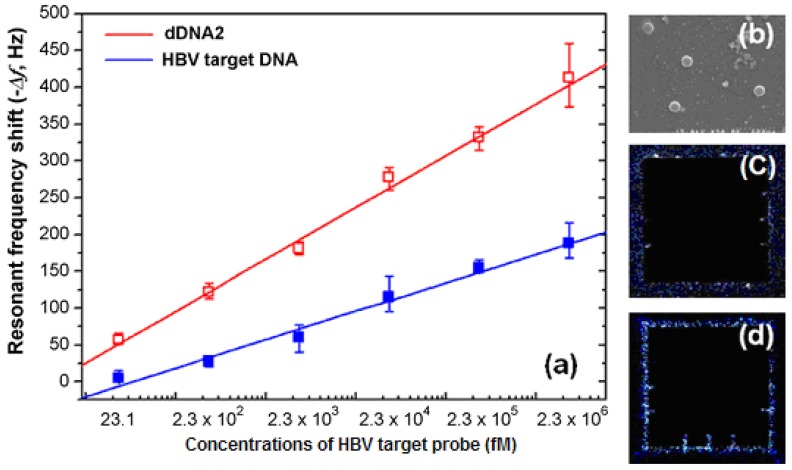
The results of the HBV DNA assay with silicon-nanoparticle (SiNP) enhanced dynamic microcantilevers: (**a**) plots of the resonant frequency shifts acquired from the HBV DNA assay and the SiNP enhanced HBV DNA assay; (**b**) SEM image of the microcantilever surface with the captured SiNPs at 2.3 pM HBV target DNA, and the fluorescent images of the microcantilevers; (**c**) top side and (**d**) bottom side at 2.3 pM (reprinted from [72] with permission).

The combination of the microcantilever with a highly specific enzyme provides a unique approach to quantifying enzyme substrates without the complication of sample labeling. The detection of hepatitis B virus DNA was achieved with a silica nanoparticle-enhanced microcantilever sensor. Gold-coated microcantilevers were functionalized with thiolated cDNA. Cr/Au layers (10 nm/50 nm) were deposited on the bottom side of the microcantilever with an e-beam evaporator. The resonant frequency was measured by an impedance analyzer and the working concentration range was approximately from 23 fM to 2.3 × 10^6^ fM (Figure 9) [72].

A class of microcantilever-based biosensors is the microcantilever immunosensors, which utilize highly selective antigen-antibody interactions. The immunosensors offer a common platform for high-throughput, multiplexed label-free analyses of biomolecules in a single step in real time, based on specific biomolecular binding, such as protein-protein binding. When antibody molecules are immobilized on one surface of a cantilever, specific binding between antibodies and antigens produces cantilever deflection. Zhao *et al.* [73] have developed a microcantilever immunosensor and an indirect competitive enzyme-linked immunosorbed assay (icELISA), which use a highly sensitive and specific monoclonal antibody (designated mAb6A9) against a copper-chelate complex. The half maximum inhibition concentration values (IC_50_) and working range, based on 10–90% inhibition of binding of mAb6A9 to Cu(II)-ethylenediamine-N,N,N',N'-tetraacetic acid (EDTA) [Cu(II)-EDTA], of the icELISA were approximately 1.8 ng·mL^−1^ and 0.2–17 ng·mL^−1^, respectively. A bending response of the microcantilever immunosensor was detectable at or below 1 ng·mL^−1^ of Cu(II)-EDTA complex. The two assays developed (microcantilever immunosensor and icELISA) were sensitive enough to monitor Cu(II) in drinking water at the legal limits set by China, the U.S. Environmental Protection Agency (EPA) and World Health Organization (WHO). The results correlated well with those obtained by graphite furnace atomic absorption spectrometry.

Among the biomolecular detection assays, the microcantilever technology can also be used for DNA-DNA hybridization detection, including accurate positive/negative detection of single base-pair mismatches [74]. This type of assay can be used to detect mutations in the DNA sequence of target DNA responsible for many cancers. The detection occurs where a single non-complementary nucleotide appears in the sequence. Single-stranded DNA (ssDNA) is immobilized on the side of the cantilever coating with gold, by means of a thiol linker bonded to one end of the ssDNA. The change in surface stress resulting from the adsorption of ssDNA on a cantilever has been found experimentally to be 30–50 mN/m. Of particular interest in current genomics research is the detection of single-nucleotide polymorphisms (SNPs). The ability to locate and characterize SNPs would aid in the early detection, diagnosis, and perhaps treatment of individuals carrying mutations causing diseases such as cystic fibrosis and thalassaemia, among others. Hansen *et al.* [34] have used hybridization-induced cantilever deflection to demonstrate that the number and location of DNA pair mismatches in a target 10-mer oligonucleotide can be discerned under high-stringency static and flow conditions using gold-coated silicon microcantilevers. Gold-coated silicon microcantilevers were functionalized with thiolated 20- or 25-mer probe DNA oligonucleotides. The magnitude of deflection was greater for the hybrids with two internal mismatches than for those with one internal mismatch, which indicates that the degree of repulsion increased due to additional base-pair mismatch.

Prostate-specific antigen (PSA) was assayed by optically detecting nanoscale motions of two-dimensional arrays of microcantilever beams [75]. Antibodies were used, covalently bound to one surface of the microcantilevers. The 2D cantilever array chip contained 80–120 reaction wells, where each well consisted of a microfluidic chamber containing 4–8 independent cantilever sensors. The surface of the microcantilever was coated with a 25-mm layer of gold, which served as the surface for immobilizing capture molecules (antibodies). A collimated laser light beam with an expanded spot size about the same size as the whole cantilever array illuminated the gold surface of the cantilevers from the glass side. The laser light reflected off each cantilever's end pad and was collected as an array of “spots” by a charge coupled device (CCD) camera. In this study, the authors used 2-[methoxypoly(ethyleneoxy)propyl]trimethoxysilane, a silane-conjugated polyethylene-glycol chain (henceforth referred as PEG-silane) for the effective surface passivation. Quantitative detection of PSA as low as 1 ng/mL was demonstrated with an array of 400 μm long cantilevers, which yielded 2 mJ/m^2^ surface stress change due to the binding of the antibody and antigen.

There have been some efforts to modify the design of the microcantilever, with the purpose of developing microcantilever-based biosensors with greater sensitivity and signal-to-noise ratio. Godin *et al.* [76] showed that the bending of the cantilever depends on the surface roughness of the gold film and gold films with larger grain sizes on the cantilever showed increased bending sensitivity. Tabard-Cossa *et al.* [77] investigated the surface stress response of micromechanical cantilever based sensors as a function of the morphology, adhesion, and cleanliness of the gold sensing surface. The surface morphology was found to influence strongly the surface stress response for molecular adsorption where ordering of the molecules is dependent on their coverage and domain size. These authors concluded that the sensitivity of bending also depends on the uniformity of the immobilization layer and cleanliness of sensing surface [77].

Ansary and Chao [78] have investigated the deflection and vibration analysis of rectangular, triangular, and step profile microcantilevers having basic and modified shapes. The surface stress induced deflection in the microcantilever is modeled by an equivalent in-plane tensile force acting on the top surface of the cantilever, in the length direction. To increase the overall sensitivity of microcantilever biosensors, both the deflection and the resonant frequency of the cantilever should be increased at the same time. The triangular and step cantilevers have better deflection and frequency characteristics than rectangular ones. The deflections usually range a few tens to a few hundreds of nanometers. Measuring deflections of this order requires extremely sophisticated readout arrangements. As the sensitivity of a microcantilever biosensor depends on the design sensitivity of the cantilever and the measurement sensitivity of the deflection measurement system, the challenge is to increase the sensitivity of a microcantilever without increasing the complexity of the deflection detection system. These authors [79] also presented a new microcantilever design with a rectangular hole at the fixed end of the cantilever, which was about 75% more sensitive than the conventional design, and the frequency analysis showed that the natural resonant frequency of the proposed design was about half the conventional frequency, *i.e.*, f_0_, _proposed_ = 0.47 f_0_,_conventional_.

The piezoresistive cantilever based biosensors are sensitive to temperature change, owing to their relatively large surface-to-volume ratio, which can have some impact on the sensor stability [80]. The geometrical parameters of this type of sensor should be optimized for optimal sensitivity. The sensitivity increases with decreasing cantilever thickness due to the reduced stiffness of the device when the ratio of piezoresistor thickness to cantilever thickness is fixed [80].

Temperature compensation has been proposed, in which a parallel microcantilever is used, with one active cantilever and another reference cantilever for noise reduction, but the different composition of the cantilevers may lead to a serious offset [81]. Yang *et al.* [81] proposed a bridge circuit composed of an active cantilever, a reference cantilever and two fixed piezoresistors for *in situ* surface stress measurements of a biochemical reaction. This circuit proved effective in offset voltage adjustment and temperature drift compensation.

Microcantilever based-biosensors can also be used in a liquid environment. Kwon *et al.* [82] reported the *in situ* real-time monitoring of a specific protein antigen-antibody interaction, using a resonating microcantilever immersed in a viscous fluid. The precise *in situ* real-time monitoring of protein-protein interactions was ascribed to the high quality factor of the resonating piezoelectric thick-film microcantilever.

## 5. Conclusions

In this review we have attempted to show the wide field of application of the atomic force microscopy (AFM) technique. Among the AFM technique, the Chemical Force Microscopy (CFM) which is based on AFM tips chemically modified with specific exposed functional groups, carefully architected to carry out a specific function in a system, appears as a powerful tool. The possibility of controlling the chemical interactions between the AFM tip and the sample can provide sensitive resources to measure and to map the surface chemistry information and to quantify the adhesive or repulsive forces associated to inorganic materials and biological samples.

The potential use of microcantilevers in a broad range of applications is also arising as a powerful tool coming from the AFM. Depending on the specific application of microcantilevers, they may be functionalized either with biological material, which converts them into microcantilever-based biosensors (MC-B) or may be functionalized with inorganic materials, which converts them into Nanomechanical Cantilever Sensors (NCS). There are two types of signals in these devices, which can be monitored simultaneously, adsorption-induced cantilever bending when adsorption is confined to one side of the cantilever and adsorption-induced vibrating frequency change due to mass loading. Depending upon the final application of the device, variety of techniques can be used to functionalize the microcantilever surface. The method used in the functionalization has a strong influence on the sensitivity and selectivity of device since they depend on the detection layer, which may be built according to principles of molecular recognition. This technology enables the development of sensors that can detect low concentrations of analytes in very small volumes of liquid or gas, with great sensitivity, reproducibility and repeatability.
